# Wood reinforcement of poplar by rice NAC transcription factor

**DOI:** 10.1038/srep19925

**Published:** 2016-01-27

**Authors:** Shingo Sakamoto, Naoki Takata, Yoshimi Oshima, Kouki Yoshida, Toru Taniguchi, Nobutaka Mitsuda

**Affiliations:** 1 Bioproduction Research Institute, National Institute of Advanced Industrial Science and Technology (AIST), Tsukuba, Ibaraki 305-8566, Japan; 2 Forestry and Forest Products Research Institute, Forest Bio-Research Center, Hitachi, Ibaraki 319-1301, Japan; 3 Technology Center, Taisei Corporation, Nase-cho 344-1, Totsuka-ku, Yokohama, Kanagawa 245-0051, Japan

## Abstract

Lignocellulose, composed of cellulose, hemicellulose, and lignin, in the secondary cell wall constitutes wood and is the most abundant form of biomass on Earth. Enhancement of wood accumulation may be an effective strategy to increase biomass as well as wood strength, but currently only limited research has been undertaken. Here, we demonstrated that OsSWN1, the orthologue of the rice NAC Secondary-wall Thickening factor (NST) transcription factor, effectively enhanced secondary cell wall formation in the Arabidopsis inflorescence stem and poplar (*Populus tremula*×*Populus tremuloides*) stem when expressed by the Arabidopsis *NST3* promoter. Interestingly, in transgenic Arabidopsis and poplar, ectopic secondary cell wall deposition in the pith area was observed in addition to densification of the secondary cell wall in fiber cells. The cell wall content or density of the stem increased on average by up to 38% and 39% in Arabidopsis and poplar, respectively, without causing growth inhibition. As a result, physical strength of the stem increased by up to 57% in poplar. Collectively, these data suggest that the reinforcement of wood by *NST3pro:OsSWN1* is a promising strategy to enhance wood-biomass production in dicotyledonous plant species.

Lignocellulose in plant secondary cell wall, composed of cellulose, hemicellulose, and lignin, constitutes wood and is the most abundant renewable carbon resource for production of biofuel and biomaterials, which could reduce the environmental impact of carbon dioxide emission. Increasing secondary cell wall accumulation is potentially beneficial for industrial uses of plant biomass and would contribute to reduction in cultivation and transportation costs, which comprise a major portion of the price for plant biomass. In addition, an increase in secondary cell wall accumulation may improve tensile properties such as stem strength and elastic modulus, which are important for mechanical performance of wood.

NAC SECONDARY WALL THICKENING PROMOTING FACTOR (NST) 1–2, and NST3/SECONDARY CELL WALL ASSOCIATED NAC DOMAIN PROTEIN1 (SND1), transcription factors containing the NAC domain, are master regulators of secondary cell wall formation except in vessel elements^1^,^2^,^3^. Overexpression of these transcription factors and their orthologues induces ectopic deposition of secondary cell wall in a variety of cell types owing to ectopic expression of genes associated with secondary cell wall biosynthesis^1^,^2^,^3^,^4^,^5^. Although these NST/SND transcription factors may be a powerful tool to enhance secondary cell wall accumulation, their overexpression driven by the CaMV 35S promoter induces abnormal organ shape and growth inhibition as a result of ectopic secondary cell wall formation and therefore proper regulation is inevitable. Yang *et al.*^6^ successfully enhanced the wall thickness of fiber cells of the inflorescence stem in an Arabidopsis (*Arabidopsis thaliana*) *c4h* mutant background by employing the *IRX8* gene promoter, which is a direct target of NST1, to express the NST1 transcription factor. In addition, Wang *et al.*^7^ reported expansion of the area of secondary cell wall formation was observed in the stem of *wrky12* knockout mutants of Arabidopsis and *Medicago truncatula.* This phenomenon was due to upregulation of NST2 and other secondary-wall-associated transcription factors, which are negatively regulated by WRKY12^7^. The similar phenotype was also observed in *athb15* knockout mutant of Arabidopsis^8^. The above-mentioned studies indicate that a proper gene regulatory system may effectively drive secondary cell wall accumulation in specific cell types without significant growth inhibition. The next challenge is to implement the strategy in plants of practical importance, such as poplar. Zhao *et al.*^4^ has recently reported that expression of shorter splicing variant of *PtrWND1B/PtVNS11* (*PtrWND1B-s*), which is one of poplar orthologues of NST transcription factors^9^,^10^, under the control of *PtrWND1B* promoter induced 37% increase of cell wall thickness in xylary fiber cells in poplar. However, no other data regarding wood biomass was provided and therefore it is still elusive if the transgenic poplar is practically beneficial for wood industry.

We previously reported that activity of the Arabidopsis *NST3* promoter is preferentially observed in fiber cells of the inflorescence stem and hypocotyl of Arabidopsis and almost no activity is observed in xylem vessels. These observations suggest that the *NST3* promoter is an excellent tool for effective secondary cell wall induction in fiber cells^2^,^11^. We further observed that overexpression of rice functional orthologue of NST1-3/SND1, *Oryza sativa* SECONDARY WALL NAC DOMAIN PROTEIN (OsSWN) 1, more strongly induced ectopic secondary cell wall deposition in epidermis cells of Arabidopsis than did Arabidopsis NSTs^12^, suggesting that *OsSWN1* is a promising candidate gene to select for induction of secondary cell wall accumulation in dicot. In the present study, we demonstrated that expression of *OsSWN1* under the control of the *NST3* promoter in Arabidopsis induces ectopic secondary cell wall formation in the pith in addition to thickening of the secondary cell wall in fiber cells. Furthermore, this enhanced secondary cell wall formation was observed in transgenic poplar (*Populus tremula*×*Populus tremuloides*) harboring the same construct without any growth retardation. These data indicated that enhancement of secondary cell wall formation by *OsSWN1* driven by the *AtNST3* promoter may be a valuable approach to boost biomass production for industrial uses of wood.

## Results

### *OsSWN1* Effectively Induced Secondary Cell Wall Formation in Arabidopsis Inflorescence Stem

**Figure 1:**
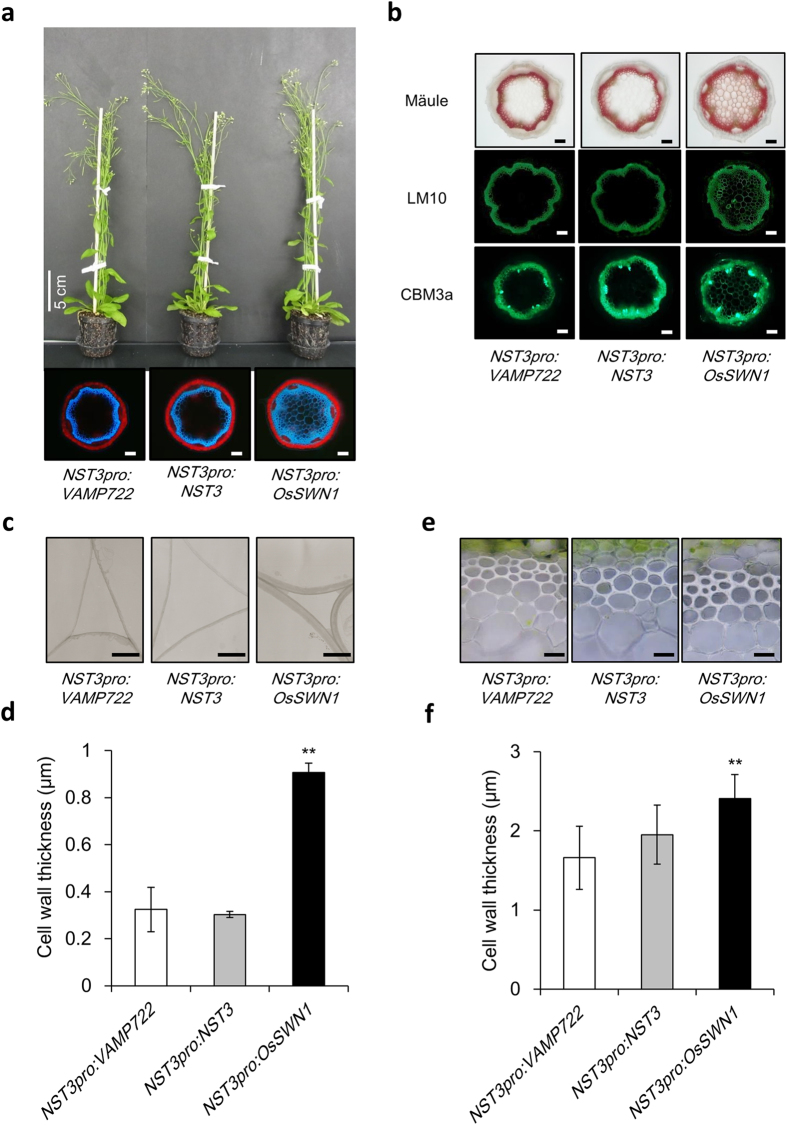
*NST3pro:OsSWN1* enhanced secondary cell wall formation (**a**) Entire appearance and cross section of inflorescence stem of *NST3pro:VAMP722* (negative control), *NST3pro:NST3*, and *NST3pro:OsSWN1* plants. The cross section was observed under UV illumination. (**b**) Cross section of inflorescence stem of the three transgenic plants, which were stained or immunolabeled with Mäule reagent, LM10, or CBM3a. (**c**) Transmission electron microscope image of pith area of the three transgenic plants. (**d**) Average cell wall thickness of the pith area of the three transgenic plants (n = 3). (**e**) Microscopic image of interfascicular fiber cells of the three transgenic plants. (**f**) Average cell wall thickness of the interfascicular fiber cells of the three transgenic plants (n = 7). Bars represent 100 μm (**a,b**), 3 μm (**c**), and 20 μm (**e**), respectively. Error bars represent SD of the biological replicates. Double asterisk(s) indicate *P* < 0.01 in Dunnett’s test when compared with negative control plant.

In the previous study^12^, we demonstrated that *OsSWN1* could restore the phenotype of *nst1-1 nst3-1* double mutant which doesn’t possess any secondary cell wall in fiber cells when its expression was driven by *NST3* promoter, which was previously shown that it is specifically active in interfascicular fiber cells of the inflorescence stem of Arabidopsis^2^. Afterwards, we noticed that some transgenic lines showed ectopic auto fluorescence of lignin in pith area of the stem under ultraviolet (UV) light (data not shown). Therefore, we considered that if this phenomenon could also occur in wild-type background, the *NST3pro:OsSWN1* construct would be valuable to increase wood production in plants of economic importance in which *nst* mutant is not readily prepared. To produce transgenic plants showing enhanced secondary cell wall accumulation in fiber cells, we expressed *OsSWN1* under the control of the *NST3* promoter. Interestingly, microscopic observation under UV illumination revealed that, in 10 out of 17 T_1_ transgenic lines, conspicuous auto fluorescence from ectopically deposited lignin was uniformly distributed in pith cell walls of the inflorescence stem in the transgenic plants expressing *OsSWN1* (Fig. 1a). The Mäule reagent also stained cell walls in the pith, providing further evidence for ectopic deposition of lignin in the transgenic plants (Fig. 1b). To examine whether this ectopic deposition of lignin was accompanied with other secondary cell wall components, transverse sections of the inflorescence stem were labelled with the LM10 monoclonal antibody for xylan or the CBM3a probe for crystalline cellulose. The presence of xylan and crystalline cellulose in the pith was clearly visualized (Fig. 1b), suggesting that secondary cell walls were ectopically deposited in the pith. Detailed observation of transverse sections by transmission electron microscopy also supported the presence of thickened secondary cell walls of the pith cells (Fig. 1c,d).

In addition, increased accumulation of secondary cell wall material in fiber cells was also observed in the transgenic plants (Fig. 1e). Detailed observation of transverse sections revealed a 45% increase in secondary cell wall thickness in fiber cells of the *NST3pro:OsSWN1* transgenic plants compared with the negative control plants in which the endosomal protein *VAMP722* gene^13^ was expressed under the control of the *NST3* promoter (Fig. 1f). Conversely, xylem vessel cell wall was slightly reduced in the transgenic plants (Fig. S1) possibly due to relative depletion of cell wall material in xylem vessel and/or negative effect of OsSWN1, which was suggested before about NST3^14^.

It should be noted that the *NST3pro:NST3* construct did not induce these excess accumulation of secondary cell walls (Fig. 1a–f), although in some lines expansion of the lignified area was observed but to a much lesser extent than that observed for *NST3pro:OsSWN1*.

### Biochemical Analysis of Cell Wall Components in the Inflorescence Stem of Arabidopsis Expressing *OsSWN1*

**Figure 2:**
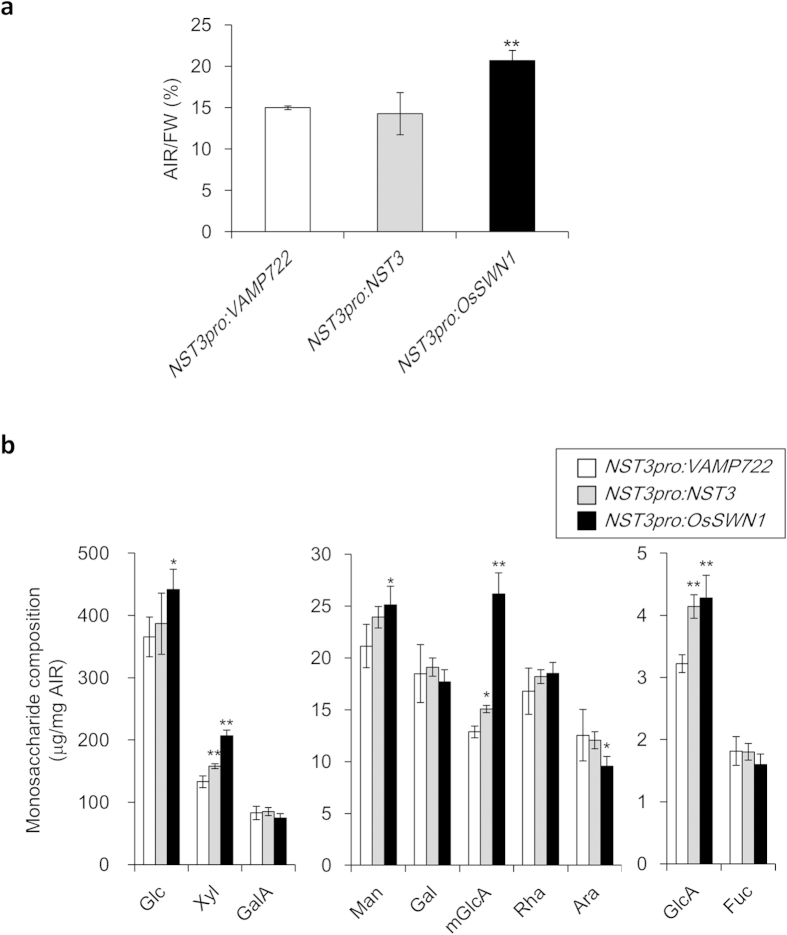
Biochemical analysis of cell wall in the transgenic plants (**a**) AIR/FW of bottom 10-cm-long inflorescence stem of *NST3pro:VAMP722*, *NST3pro:NST3*, and *NST3pro:OsSWN1* plants (n = 4). (**b**) Monosaccharide composition of the three transgenic plants. Glc, D-glucose; Xyl, D-xylose; GalA, D-galacturonic acid; Man, D-mannose; Gal, D-galactose; mGlcA, 4-*O*-methyl–D-glucuronic acid; Rha, L-rhamnose; Ara, L-arabinose; GlcA, D-glucuronic acid; Fuc, L-fucose. Error bars represent SD. Single and double asterisk(s) indicate *P* value < 0.05 and < 0.01, respectively in Dunnett’s test when compared with negative control plant.

We investigated the accumulation of chemical compounds in the inflorescence stem of Arabidopsis expressing *OsSWN1*. First, we compared the amount of alcohol insoluble residue (AIR). The amount of AIR in the *OsSWN1*-expressing stem was 38% higher than that in the stem of the negative-control plants, whereas no significant difference in percentage stem AIR was observed between the negative-control plants and plants expressing *NST3* under the control of the *NST3* promoter (Fig. 2a). The amounts of glucuronic acid (GlcA), 4-*O*-methyl glucuronic acid (mGlcA), and xylose (Xyl), which were mostly derived from secondary cell walls, and glucose (Glc) and mannose (Man), which were derived from cellulose, mannan, and glucomannan in primary and secondary cell walls, in the AIR of *OsSWN1*-expressing stems were higher than those in stems expressing *VAMP722* and *NST3* (Fig. 2b). In contrast, the content of arabinose (Ara), which is associated with primary cell walls, was reduced, although the contents of galacturonic acid (GalA), galactose (Gal), and fucose (Fuc) were not altered (Fig. 2b). These data indicated that secondary cell wall components accumulated to higher amounts in the inflorescence stem of Arabidopsis expressing *OsSWN1* than in the stem of plants expressing *VAMP722* or *NST3*.

### Strong Transactivation Ability of *OsSWN1* Upregulates Genes Associated with Secondary Cell Wall Formation

**Figure 3:**
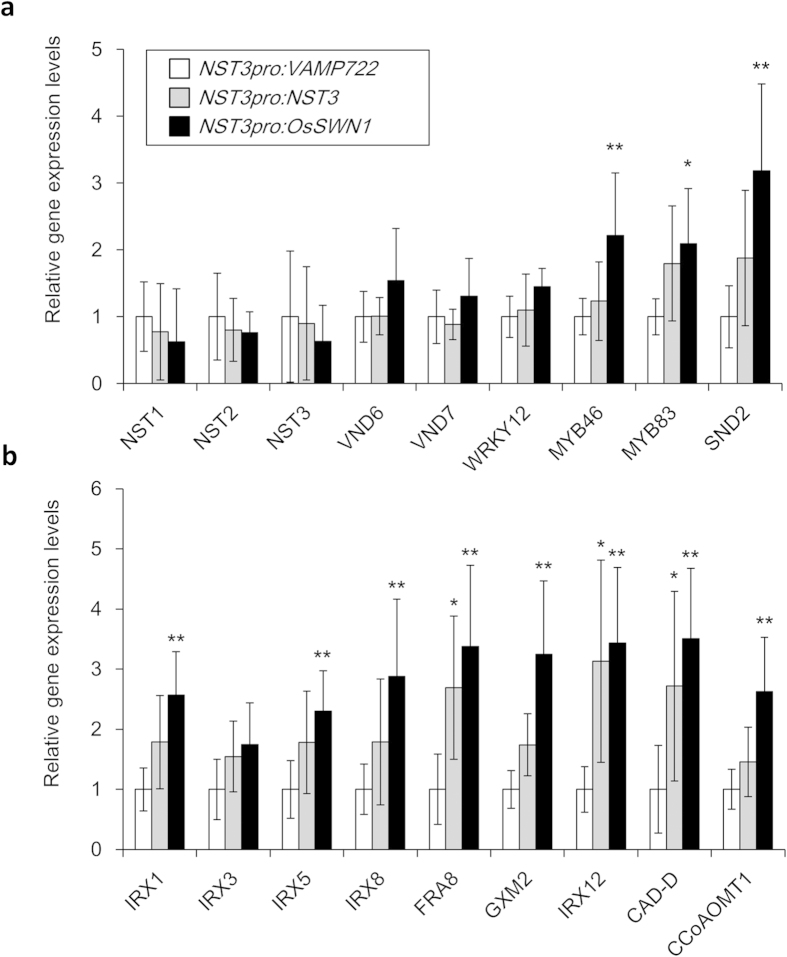
Expression of genes related to secondary cell wall formation in the transgenic plants. Relative expression levels of genes related to secondary cell wall formation. Transcription factor genes (**a**) and enzymatic genes (**b**) are separately shown. Relative expression of each gene in the negative control plant was set to 1. Error bars represent SD of six biological replicates. Single and double asterisk(s) indicate *P* value < 0.05 and < 0.01, respectively in Dunnett’s test when compared with the negative control plant.

To investigate the regulatory mechanism of the enhanced secondary cell wall formation in *OsSWN1*-expressing stems, we analyzed the expression level of genes associated with secondary cell wall formation in the transgenic plants. As shown in Fig. 3a, Expression of downstream transcription factors, such as MYB46, MYB83 and SND2, was enhanced compared with the negative-control and *NST3pro:NST3* plants, whereas expression of *WRKY12*, which is a negative regulator of NST2, was neither induced nor repressed (Fig. 3a). As a result, expression of most enzymatic genes associated with cellulose, xylan, and lignin biosynthesis was induced in the *NST3pro:OsSWN1* plants (Fig. 3b). These genes were also induced in the *NST3pro:NST3* transformant compared with the negative-control plant but to a lesser extent than in the *NST3pro:OsSWN1* plants (Fig. 3b). This finding may reflect the expansion of the lignified area to pith observed in some *NST3pro:NST3* lines.

**Figure 4:**
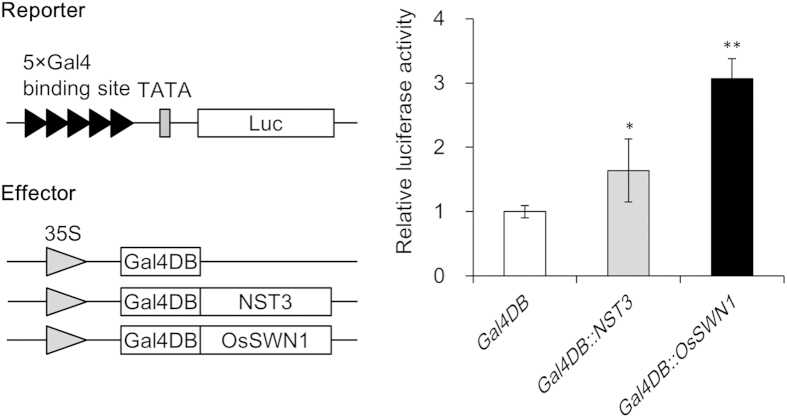
Reporter effector analysis of OsSWN. Schematic diagram of the reporter and effector constructs are shown in left panel. Right panel indicates relative reporter activity when indicated effector was co-transformed (n = 4). Error bars represent SD of technical replicates. Single and double asterisk(s) indicate *P* value < 0.05 or 0.01 in Dunnett’s test when compared with negative control (GAL4DB).

Next, we examined the transactivation ability of OsSWN1 in a transient reporter–effector experiment. In this experiment, we employed NST3 and OsSWN1 fused to DNA-binding domain of yeast transcription factor GAL4 as effectors (GAL4DB:NST3 and GAL4DB:OsSWN1) and transformed into leaf protoplast together with reporter construct containing luciferase driven by promoter with repeated GAL4-binding sequence (Fig. 4). We found by this experiment that GAL4DB:OsSWN1 induced higher reporter activity than GAL4DB:NST3, suggesting that the transactivation ability of OsSWN1 was higher than that of NST3 (Fig. 4). However, this doesn’t directly explain the ectopic secondary cell wall deposition in pith of the *NST3pro:OsSWN1* plants. We therefore carefully examined the promoter activity of *NST3* in stem cross section again by using *NST3pro:GUS* plants and found that GUS activity was faintly observed in pith (Fig. S2a–c). Furthermore, RT-PCR experiment using isolated cells by laser-micro-dissection revealed that *NST3* is faintly expressed in pith while expression of *SND2* and *MYB63*, which were known to be involved in secondary cell wall formation and are highly expressed in fiber cells^15^,^16^, was not detected (Fig. S2d), suggesting that the detected expression of *NST3* in pith was unlikely to be contamination from other tissues. From these data, we concluded that the original very weak *NST3* promoter activity in pith is boosted by the stronger transactivation ability of OsSWN1 than that of NST3, leading to the over/ectopic-accumulation of secondary cell wall components in *NST3pro:OsSWN1* transformants in pith.

### Enhancement of Secondary Cell Wall Deposition in Woody Tissue of Poplar

Before applying the OsSWN1-expressing approach to poplar, we first examined the effect of fusion to OsSWN1 of the strong transactivation domain of VP16 and employment of the newly developed HSP terminator^17^. The AIR content of the *NST3pro:OsSWN1-VP16* plant was 75% higher than that of the control plant (Fig. S3), while a 38% increase was observed in case of no fusion of VP16 and with NOS terminator, suggesting that VP16 fusion and/or the HSP terminator boosted the effect. Therefore, we decided to apply the *OsSWN1-VP16* fusion construct with the HSP terminator for transformation of poplar. Even in young seedlings of transgenic poplar grown in culture media (plant height less than 15 cm), secondary cell wall over-accumulation was observed as shown by staining of lignin with phloroglucinol and scanning electron microscopic observation (Fig. S4a–d). The average AIR content of five independent plants was 31% higher than that of control plants, thus indicating that secondary cell wall formation was enhanced (Fig. S4e).

**Figure 5:**
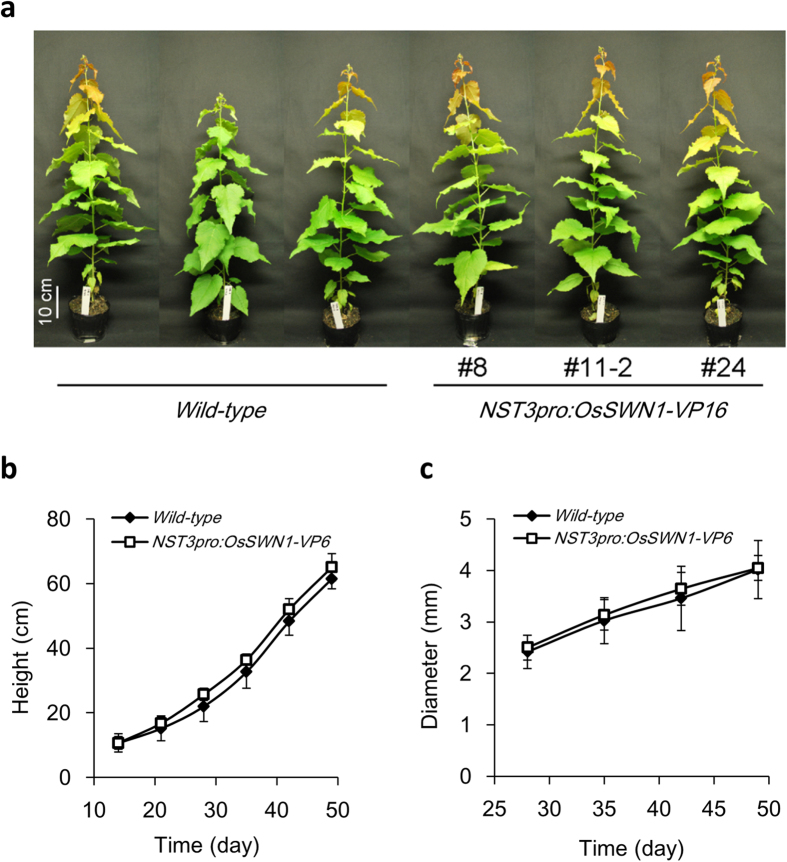
Growth of transgenic *NST3pro:OsSWN1-VP16* poplar (**a**) Entire appearance of wild-type poplar and *NST3pro:OsSWN1-VP16* poplar grown for 49 days in soil. (**b,c**) Change of average plant height (**b**) and average stem diameter (**c**) of wild-type (n = 3) and of transgenic poplar (n = 5) for 49 days after transferring plants onto soil. Error bars represent SD of biological replicates.

**Figure 6:**
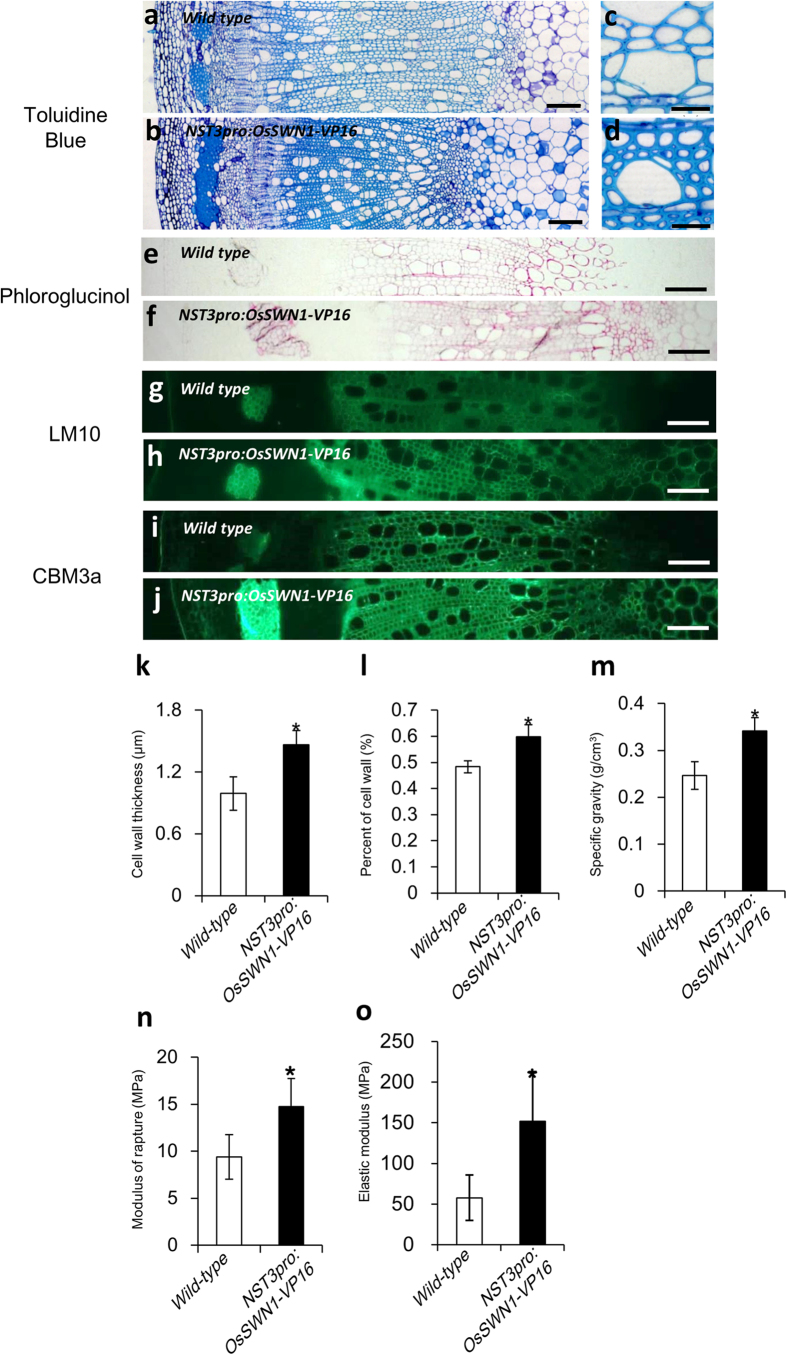
*NST3pro:OsSWN1-VP16* enhances wood accumulation in poplar (**a–j**) Stem cross section of wild type (**a,c,e,g,i**) and the *NST3pro:OsSWN1-VP16* (**b,d,f,h,j**) plant stained with toluidine blue (**a–d**), phloroglucinol (**e,f**), LM10 (**g,h**) and CBM3a (**i,j**). Bars represent 100 μm (**a,b**), 20 μm (**c,d**), and 100 μm (**e–j**). (**k,l**) Average cell wall thickness of fiber cell (**k**) and cell wall area in xylem (**l**) of wild-type (n = 3) and *NST3pro:OsSWN1-VP16* plants (n = 5). (**m–o**) Average specific gravity (**m**), module of rapture (**n**), and elastic modulus (**o**) of wild type (n = 3) and *NST3pro:OsSWN1-VP16* (n = 5) stem segments. Error bars represent SD of biological replicates. Asterisk indicates *P* < 0.05 in Welch’s t-test when compared with wild-type.

Next, the transgenic poplar plants were transplanted into soil and cultivated for 49 days, at which stage the extent of secondary cell wall enhancement and growth rate were examined (Fig. 5). No growth inhibition was observed among the transgenic plants (Fig. 5a–c). However, obvious thickening of the secondary cell wall was observed in xylem cells and phloem fiber cells but not in xylem vessels of the transgenic plants (Fig. 6a–d). In addition, staining of lignin with phloroglucinol and immunolabeling with LM10 and CBM3a revealed that the three major components of secondary cell walls, namely lignin, xylan, and crystalline cellulose, were deposited in cell walls of the pith as well as xylem fibers and phloem fibers of transgenic poplar (Fig. 6e–j). Further detailed image analysis revealed an 48% increase in cell wall thickness (Fig. 6k) and 24% increase in total cell wall area in the xylem (Fig. 6l). As a result, the average density of absolute-dried stem segments from five independent events (plants) was 39% higher than that of control plants (Fig. 6m). Furthermore, the average physical strength, which was measured as the breaking force per cross-sectional area, and elastic modulus of the stem of these five plants was 57% and 160% higher than that of control plants, respectively (Fig. 6n,o). These data indicated that reinforcement of the secondary cell wall by OsSWN1-VP16 under the control of the *NST3* promoter effectively improved wood productivity and quality, in terms of cell wall density and stem strength, in poplar.

## Discussion

The most successful example of enhanced secondary cell wall deposition was the artificial positive feedback loop (APFL) system in Arabidopsis reported recently^6^. It was carried out using *IRX8pro:NST1* and successfully enhanced secondary cell wall thickening in fiber cells of Arabidopsis without any obvious impairment of growth. However, the utility of the APFL system in plants of economic importance remains to be examined.

The present study demonstrated that expression of *OsSWN1* under the control of the *NST3* promoter is effective to enhance secondary wall formation in fiber cells of Arabidopsis and poplar without notable growth inhibition (Figs 1a and 5). How this ideal increase is accomplished in terms of energy/carbon source supply is important issue for biomass production but still elusive. We consider there is a hint in the *nst1-1 nst3-1* double mutant which doesn’t produce any secondary cell walls in fiber cells^2^. Even though the double mutant doesn’t produce secondary cell walls which usually occupy around half of plant dry weight^11^, the growth speed was not accelerated in the double mutant^2^, suggesting that the double mutant may attenuate photosynthetic activity due to less sink demand. This observation lets us hypothesize that photosynthetic activity of the *NST3pro:OsSWN1* plants is enhanced to some extent to cope with increased sink demand. Otherwise, resource allocation may be changed in the *NST3pro:OsSWN1* plants. Further study is needed to clarify this important point for biomass production and probably, increase of photosynthetic activity by genetic manipulation would be required to achieve further increase of biomass production.

It should be noted that the effect of cell wall increase tends to be less pronounced in poplar than in Arabidopsis (for instance, compare Figs S3 and S4e) while increase of cell wall thickness looks similar in poplar and Arabidopsis (compare Figs 1f and 6k). This less effect in poplar could be due to larger amount of cell wall and xylem tissues in polar than Arabidopsis. Otherwise, the difference of ability to induce secondary cell wall formation between OsSWN1 and poplar orthologues could be smaller than that between OsSWN1 and Arabidopsis NSTs. If poplar NST orthologues are strong enough, the effect of OsSWN1 introduction could be limited. Further case study is needed to demonstrate the utility of OsSWN1 in other plants of economic importance.

In the transgenic plants, enhancement of secondary cell wall formation was not observed in xylem vessels of both Arabidopsis and poplar (Figs S1 and 6c,d). These data confirm the utility of the *NST3* promoter to enhance secondary cell formation in fiber cells and also suggest that the *cis-*elements for fiber-specific expression, which should reside within the *NST3* promoter, are well conserved between Arabidopsis and poplar. Interestingly, a pronounced effect was also observed in the pith of Arabidopsis (Fig. 1a–c) and poplar (Fig. 6c,d), and in phloem fibers in poplar (Fig. 6a,b,g–j). These data suggest that the Arabidopsis *NST3* promoter shows a certain level of activity in these tissues as well as fiber cells as supported by the promoter:GUS experiment and RT-PCR experiment in Arabidopsis (Fig. S2).

Recently, the WRKY12 transcription factor was reported to be an upstream negative regulator of NST2^7^. In the stem of *wrky12* knockout mutants of Arabidopsis and *Medicago truncatula*, the area of secondary cell wall formation was enlarged through upregulation of *NST2* and other secondary-cell-wall-associated transcription factors^7^. The ectopic deposition of secondary cell wall in the pith of *NST3pro:OsSWN1* plants is similar to the phenotype of the *wrky12* mutant. However, in the transgenic plants, neither suppression of *WRKY12* expression nor induction of *NST2* expression was observed. We consider that the conspicuous phenotype of the transgenic plants was caused by the strong transactivation ability of OsSWN1 as shown in Figs 3 and 4. In general, NAC family proteins, which include NSTs and OsSWNs, contain a DNA-binding motif, the NAC domain, at the N-terminal end and a non-conserved region involved in transactivation at the C-terminal end^3^,^18^. While the NAC domain at the N terminus of NSTs and OsSWNs show high homology, the C-terminal structure of the proteins is not highly conserved. In most monocotyledonous NST/SND orthologues, the C-terminal protein sequence is 30–60 amino acids longer than that of NST3 and may reinforce the transactivation ability of these proteins (Fig. S5). In addition, an inhibitory protein variant of the NST orthologue is known in poplar, which is derived from a splicing variant of the same locus^19^. Such an inhibitory protein may be non-functional against OsSWN1 from gramineae.

In summary, we successfully enhanced secondary cell wall accumulation in fiber and pith cells in Arabidopsis and poplar without impairment of growth. This enhancement of secondary cell wall accumulation in poplar effectively improved the wood quality in terms of cell wall density and stem strength. Transformation of poplar will be of considerable benefit for the wood industry by increasing productivity and improving wood quality. We consider that the strategy reported herein is an innovative tool with potential to enhance future uses of wood, such as biofuel and biomaterial production.

## Methods

### Plant Materials and Growth Conditions

*Arabidopsis thaliana* ecotype Columbia-0 was grown in soil at 22 °C under a 16 h/8 h (light/dark) photoperiod (photon flux density [PFD] 60–70 μmol m^−2^ s^−1^) for 2 months and transformed using the floral-dip method. Transgenic T_1_ seeds were selected on 1/2 Murashige and Skoog agar medium with hygromycin and grown for 3 weeks. The hygromycin-resistant transgenic plants were transferred to soil and further harvested for 4–5 weeks. The basal 10-cm segment of the inflorescence stem was used for chemical analyses of cell walls. *Populus tremula*×*Populus tremuloides* (wild type clone T89) was transformed by *Agrobacterium*-mediated transformation^20^. Cultured plants were transplanted to soil mix (3:1 fertilized peat moss:vermiculite) and grown at 18 °C under long-day conditions (18 h/8 h [light/dark]; PFD 300 μmol m^−2^ s^−1^). Tree height and stem diameter at 10 cm above the soil surface was measured weekly.

### Vector Construction

*NST3pro:OsSWN1* and *NST3pro:VAMP722* was constructed by inserting the guanine-added *OsSWN1* or *VAMP722* coding sequence into the *Sma*I site of the pNST3_NOS_Entry vector. The pNST3_NOS_Entry vector was constructed by ligating the amplified *NST3* promoter (ca. 3 kb) into the *Asc*I/*Bam*HI site of the pNOS_Entry vector. The pNOS_Entry vector was constructed by inserting artificial multi-cloning sites into the *Hin*dIII/*Sal*I-digested p35SG_v2 vector. The p35SG_v2 vector was generated by mutating the *Hin*dIII and *Eco*RI sites, which reside outside the *att*L sites of the p35SG vector^21^. *NST3pro:OsSWN1-VP16* was constructed by inserting the guanine-added *OsSWN1* coding sequence into the *Sma*I site of the pNST3_VP16_HSP_Entry vector. The pNST3_VP16_HSP_Entry vector was generated by ligating the amplified *NST3* promoter (ca. 3 kb) into the *Asc*I/*Bam*HI site of the pVP16_HSP_Entry vector. The pVP16_HSP_Entry vector was generated by ligating the *Sma*I/*Eco*RI-excised fragment from the p35SVP16HSPG vector into the above-mentioned pNOS_Entry vector. The p35SVP16HSPG vector was formed by replacing SRDX of the p35SSRDXHSPG vector^21^ with VP16 using the *Sma*I and *Sal*I sites. Both *NST3pro:OsSWN1:NOSter* and *NST3pro:VAMP722:NOSter* were transferred to the pBCKH T-DNA vector^22^ while *NST3pro:OsSWN1-VP16:HSPter* was transferred to the pBCKK T-DNA vector^22^ by the Gateway LR reaction. All oligonucleotides used for these plasmid constructions are listed in Table S1.

### Microscopic Observation

The 2–3-cm basal portion of the inflorescence stem of transgenic Arabidopsis was embedded in 5% agar and micro-slice sections (50 μm) were prepared with a vibrating microtome (HM-650 V, Thermo Fisher Scientific, Inc., MA, USA) and fixed in FAA solution (50% ethanol, 10% formaldehyde and 5% acetic acid). The auto fluorescence of lignin in the sections was observed with the following specifications (excitation filter: 365-nm short pass, dichroic mirror: 395 nm; emission filter: 400-nm long pass). All light and fluorescence images were captured with Axioscop2 Plus software (Zeiss Inc., Oberkochen, Germany). For anatomical assays of poplar, stem samples were collected from 20th internode and fixed in FAA solution. The samples were dehydrated through a 50%, 70%, 90%, 95%, and 100% ethanol series, and then embedded in LR White (TAAB, Aldermaston, UK). Transverse sections of 2-μm thickness were cut using a Leica Jung RM2055 microtome (Leica Microsystems, Wetzlar, Germany), then subjected to toluidine blue staining, lignin staining and immunolabeling (described below). The sections stained with 0.5% toluidine blue solution were visualized using a Leica DMR microscope with a Leica DFC290 (Leica Microsystems). Cell wall thickness for 100 fiber cells and cell wall percentage for eight xylem files (340 μm × 340 μm) per each event was measured using ImageJ software.

### Lignin Staining and Immunolabeling of Stem Micro-sliced Sections

The fixed Arabidopsis micro-sliced sections of stems fixed in FAA solution were rinsed three times with ultrapure water. For visualization of lignin, sections were stained with 1% KMnO_4_ for 5 min, then washed with ultrapure water three times. After incubation in 10% HCl for 5 min, the sections were washed with ultrapure water three times and mounted in 1.5 M Na_2_CO_3_. Staining with phloroglucinol was performed as described previously^12^. For immunolabeling, micro-sliced stem sections were incubated with blocking buffer (Blocking One, Nacalai tesque, Inc., Kyoto, Japan) for 60 min at 10 °C with 100 rpm. The sections were immunolabeled with CBM3a (1:100, Plant Probes, Inc., UK) or LM10 (1:20, Plant Probes, Inc.) prepared with TBS buffer containing 20 mM Tris-HCl (pH 7.5) and 150 mM NaCl for 60 min. After three washes with TBS-T buffer containing 20 mM Tris-HCl (pH 7.0), 150 mM NaCl, and 0.2% Tween-20, secondary antibody (1:100, Anti-His-tag-Alexa Fluor® 488 [Medical and Biological Laboratories, Inc.] for CBM3a or 1:100 Anti-goat IgG mAb-Alexa Fluor 488 [Plant Probes, Inc.] for LM10) prepared with TBS buffer was reacted for 60 min. Finally, the section was rinsed with TBS-T buffer and the fluorescence was observed with fluorescence microscopy (Zeiss Inc.)

### Transmission Electron Microscopy

The basal portion of 7-week-old inflorescence stems of transgenic plants was fixed in 2% (v/v) glutaraldehyde in 0.05-mM potassium phosphate buffer (pH 7.0). Ultrathin sections (ca. 100 nm) were prepared as previously described^23^. The sections were stained with uranyl acetate and lead citrate, and observed under a Hitachi H-7500 transmission electron microscope (Hitachi Science System, Inc., Japan) at an accelerating voltage of 100 kV.

### Monosaccharide Composition Analysis

Preparation of the AIR from the inflorescence stem was described previously^11^. Air-dried AIR segments were weighed and powderized using stainless steel beads (6 mm; Biomedical Science, Inc., Japan) and three zilconia beads (3 mm; Nikkato, Inc., Japan) with a ShakeMaster NEO grinder (Biomedical Science Inc.). Starch was degraded with amylase solution containing 100 U ml^−1^ amylase (Megazyame Inc.) and 0.33 U ml^−1^ amyloglucosidase (Megazyme, Inc.) at 37 °C for 18 h. The remaining residue was washed three times with ultrapure water and three times with 100% ethanol, then dried at 65 °C overnight. The purified cell wall residues were hydrolyzed using a two-step sulfuric acid method as described previously^11^. The monosaccharides in the hydrolysate were labelled with ABEE reagent and quantified using an UPLC system equipped with a fluorescence detector^24^.

### Quantitative RT-PCR Analysis

Total RNA was extracted from 10-cm-long piece of inflorescence stem of T_1_ Arabidopsis lines using the RNeasy Plant Mini Kit (Qiagen, Inc., Hilden, Germany). First-strand cDNA was synthesized using the PrimeScript RT Reagent Kit (Takara-Bio, Inc., Otsu, Japan). Quantitative RT-PCR analysis was carried out with the SYBR Green PCR Master Mix (Life Technologies, Inc.) using an ABI 3700 Real-Time PCR system (Life Technologies, Inc.). The expression level of each gene was calculated using the absolute quantification method and expressed as the relative value compared with the average expression level of *UBQ1*, *ACT2*, and *PP2AA3*. Primers used for qRT-PCR are listed in Table S1.

### Transactivation Ability Analysis

Protoplasts were isolated from peeled rosette leaves from 3- to 4-week-old *Arabidopsis* plants using the Tape—Arabidopsis Sandwich method^25^ and prepared as described previously^12^. The reporter construct containing firefly luciferase driven by the promoter containing 5 × Gal4 DNA-binding sequence^26^ was transfected into the protoplast using the polyethylene glycol method as described previously^12^ together with the effector construct containing NST3 or OsSWN1 fused with the Gal4 DNA-binding domain driven by the CaMV 35 S promoter. The modified *Renilla* luciferase gene driven by the CaMV 35 S promoter (phRLHSP) was also co-transfected as an internal control. The transfected protoplasts were incubated at 22 °C for 16–18 h in the dark. The dual-luciferase assay was carried out using the Pikka Gene Dual Assay Kit (Toyo Ink, Inc., Tokyo, Japan). The reporter activity was normalized with the activity of the *Renilla* luciferase gene and expressed as relative luciferase activity.

### Laser micro dissection

The fresh basal 5-cm segment of inflorescence stems grown for 6 weeks were embedded in SCEM gel (Leica Microsystems) in cold isopentane and the frozen-stem was sliced using a Cryostat (CM3050 S, Leica Microsystems) as described by Kawamoto^27^. The pith cells, interfascicular fiber cells, and xylem cells of inflorescence stem were dissected using a Leica LMD6500 system. The total RNA from each was extracted using a PicoPure RNA Isolation Kit (Arcuturus Inc., CA, USA).

### Specific Gravity

A stem segment from the 25th internode was sampled and the xylem tissue was obtained by peeling off the bark. The xylem was filled with ultrapure water under reduced pressure. The xylem volume was measured by Archimedes’ principle at 20 °C^28^. The samples were dried in an oven at 105 °C for 72 h, then the dry weight was measured. The specific gravity was calculated as the ratio of xylem dry weight to volume.

### Measurement of tensile properties

Air-dried poplar stem segments ca. 1-cm long were subjected to bending test with AC-10 kN-CM instrument (T.S.E, Inc., Yokohama, Japan) as follows. In brief, the stem segment was placed bridging two metal supports separated by 5.5 mm. The center of the stem segment was pressed with a metal stick and the distortion and loading force were monitored until the stem ruptured. The breaking force was then divided by the cross-sectional area calculated from the average diameter measured in two orientations. Elastic modulus was calculated using these data according to previous study^29^.

## Additional Information

**How to cite this article**: Sakamoto, S. *et al.* Wood reinforcement of poplar by rice NAC transcription factor. *Sci. Rep.* **6**, 19925; doi: 10.1038/srep19925 (2016).

## Supplementary Material

Supporting information
