# Supplementary material for: Wood reinforcement of poplar by rice NAC transcription factor
Source: Sci Rep. 2016 Jan 27;6:19925. doi: 10.1038/srep19925 (PMC4728686; doi:10.1038/srep19925)
Supplement: Supporting information [file srep19925-s1.pdf]

Wood reinforcement of poplar by rice NAC transcription factor

Shingo Sakamoto, Naoki Takata, Yoshimi Oshima, Kouki Yoshida, Toru Taniguchi, Nobutaka Mitsuda

Supporting information

1 supplementary table, 5 supplementary figures

Table S1. Oligonucleotides used in this study

Name	Sequence	Purpose
OsSWN1F	GATGAGCATATCGGTGAACGGGCAGTCGGT	Amplification of guanine-added OsSWN1 coding sequence
OsSWN1R	TTATACGTTATTCATGGTCGTCAAGTCTGC	Amplification of guanine-added OsSWN1 coding sequence
NST3proF_Asc	TAACAAGGCGCGCCGATTCTACACATTCACAAAGTTTACTAC	Amplification of NST3 promoter region
NST3proR_Bam	ATTCCTGGATCCATTAACGAAGATAGCAATATATTTTTGGG	Amplification of NST3 promoter region
MCS_F	AGCTTAATTAAGGCGCGCCGAGCTCCACCGCGGTGGCGGCCGCTCTAGAACTAGTGGATCCCCCGGGGGT[illegible]	Inserting multi-cloning site
MCS_R	TCGACGGTACCCCCGGGGGATCCACTAGTTCTAGAGCGGCCGCCACCGCGGTGGAGCTCGGCGCGCCTTA[illegible]	Inserting multi-cloning site
VP16_F	GGGGCCCCCCCGACCGATGTCAGCC	Amplification of VP16 domain
VP16_R_Sal	AAAAATTTGTCGACCTACCCACCGTACTCGTCAATTC	Amplification of VP16 domain
UBQ1-266F	TGAGCCTTCCTTGATGATGCT	qRT-PCR analysis
UBQ1-329R	GCACTTGCGGCAAATCATCT	qRT-PCR analysis
ACT2F	ACATTGTGCTCAGTGGTGGA	qRT-PCR analysis
ACT2R	GAGATCCACATCTGCTGGAAT	qRT-PCR analysis
PP2AA3rtF	GACCAAGTGAACCAGGTTATTGG	qRT-PCR analysis
PP2AA3rtR	TACTCTCCAGTGCCTGTCTTCA	qRT-PCR analysis
NST1rtF	CGGACAATCTCAAGTGCCTCC	qRT-PCR analysis
NST1rtR	TGTCCTTGTGGCTAAAGAAATACCAGTCG	qRT-PCR analysis
NST2rtF	ATCCCACCGGGACTAGAACCA	qRT-PCR analysis
NST2rtR	GTCTTTCGCATCCCGATTCTATCACC	qRT-PCR analysis
NST3rtF	AGCTCCTCACGGTCAGAAATC	qRT-PCR analysis
NST3rtR	TCTTCCTGAACACTCGACATACCA	qRT-PCR analysis
VND6rtF	GGTGGTTTGTCGCGCTTTCA	qRT-PCR analysis
VND6rtR	CATTTGCTGCGTGAACCAGTTAGA	qRT-PCR analysis
VND7rtF	GCGAGGTGTAAACTAGGGTATGA	qRT-PCR analysis
VND7rtR	GTAGGGTACTTCCTGTCCTTATGA	qRT-PCR analysis
WRKY12rtF	GCCTTCACCCCAGGAGTTAT	qRT-PCR analysis
WRKY12rtR	GGTTGTGACGACCTTCGTAAGTA	qRT-PCR analysis
MYB46rtF	CATCCTCGGCAACAGGTGGT	qRT-PCR analysis
MYB46rtR	TGGAGGTATCGGACATCTTCTTTA	qRT-PCR analysis
MYB83rtF	TCGCCTTCGCTGGATCAATTAC	qRT-PCR analysis
MYB83rtR	GCTATTTGAGACCACCTGTTACCA	qRT-PCR analysis
SND2rtF	GTCTTGGAGCATCTTGAAGGC	qRT-PCR analysis
SND2rtR	CGTCCTTGTTCACTCCTGGCA	qRT-PCR analysis
IRX1rtF	GTCACATCGAAAACCGCAGATGAT	qRT-PCR analysis
IRX1rtR	AAGTGACGTCGGAGGGATCAA	qRT-PCR analysis
IRX3rtF	GGCAAACTCAAGTGGCTTGAGCG	qRT-PCR analysis
IRX3rtR	TAACTCCGCTCCATCTCAATTCC	qRT-PCR analysis
IRX5rtF	CGGCAAGTTCATCATTCCTACGA	qRT-PCR analysis
IRX5rtR	CACTCCACCGGAGTTCTAAGA	qRT-PCR analysis
FRA8rtF	GCTTCCACACCATGGAGGAT	qRT-PCR analysis
FRA8rtR	GTTCTCTACCTCTTGGCATGGA	qRT-PCR analysis
IRX8rtF	CTTCTCCGATTCGTCTTTGTTCTC	qRT-PCR analysis
IRX8rtR	CAAGCAAGCAAGAGAGGAGCAA	qRT-PCR analysis
GXM2rtF	CAGGGAGGATGAGCGCTATT	qRT-PCR analysis
GXM2rtR	TCAGTCTCCCCACCGTCATA	qRT-PCR analysis
IRX12rtF	CGGGGTTTGGTTCATGCATTGT	qRT-PCR analysis
IRX12rtR	ATCCTTAGGCGGCGGCAAAA	qRT-PCR analysis
CAD-DrtF	CTCTGCTTATGCTTGGGAGGA	qRT-PCR analysis
CAD-DrtR1	CGAGTCTCTCAAACGCAGTGTTA	qRT-PCR analysis
CCoAOMT1rtF	CTCCCTGAAGACGGCAAAATTC	qRT-PCR analysis
CCoAOMT1rtR	CCGGCTTTCTCAATGATCGGTAAA	qRT-PCR analysis

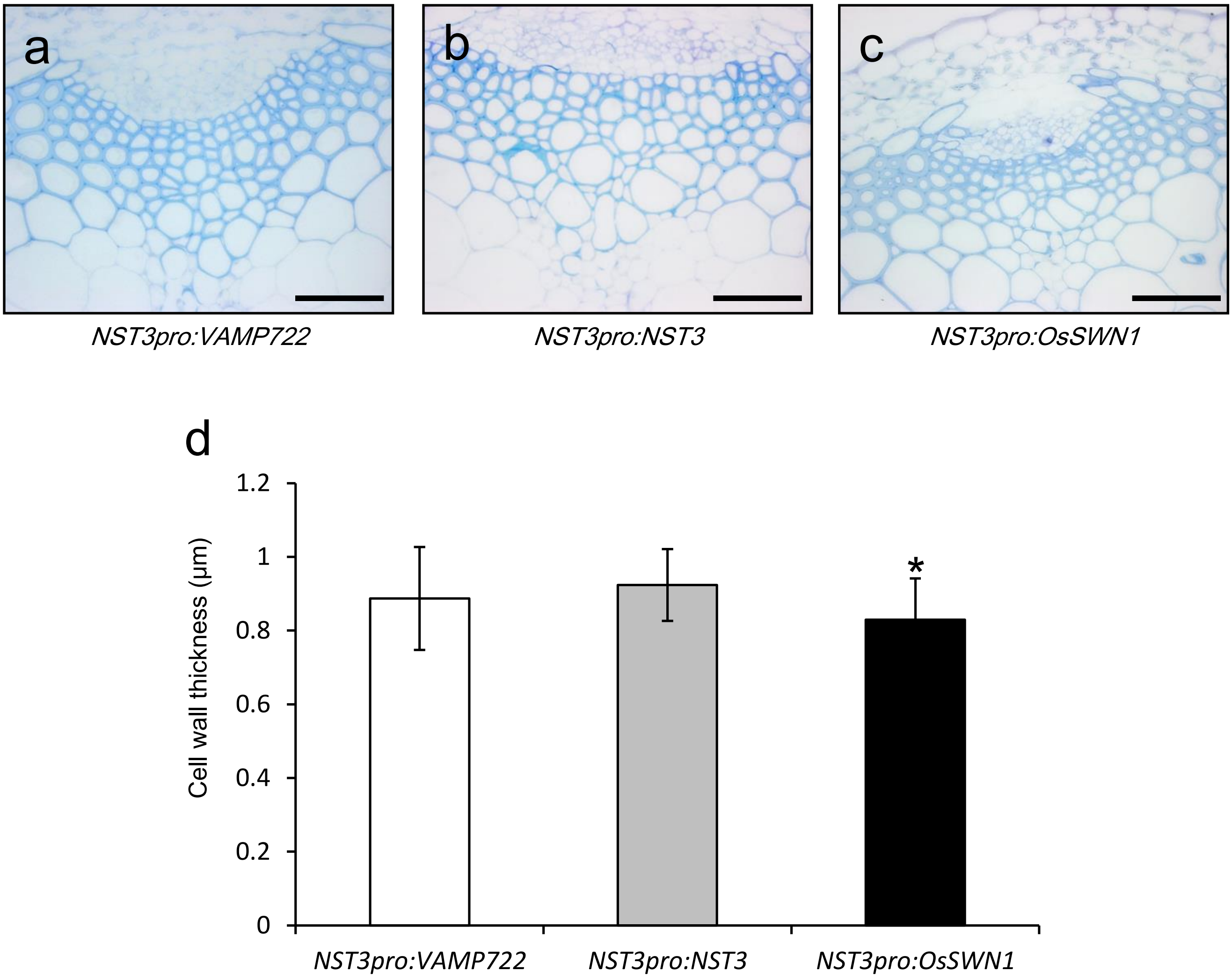

Figure S1. Secondary cell wall formation of xylem vessels in *NST3pro:OsSWN1* plants. (a-c) Cross sections of inflorescence stems of transgenic plants expressing *VAMP722*(a), *NST3* (b), and *OsSWN1*(c) driven by *NST3* promoter stained with toluidine blue. Bars represent 50 µm. (d) Cell wall thickness of xylem vessel was measured by ImageJ software. Error bars represent SD (n=60). Asterisk indicates a statistically significant difference (Dunnett's test, $P<0.01$) from *NST3pro:VAMP722* plant.

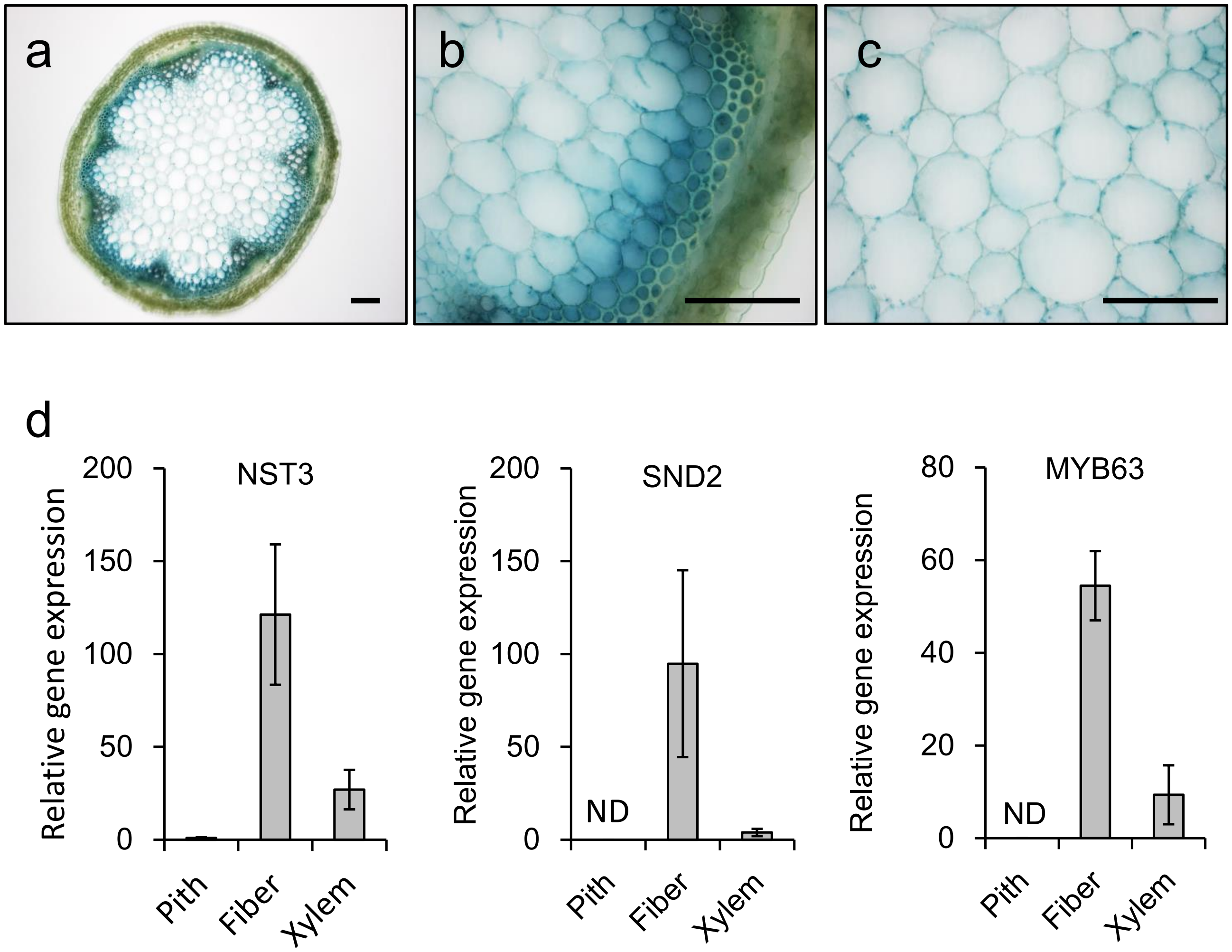

Figure S2. *NST3* is weakly expressed in pith. (a-c) GUS staining of transgenic Arabidopsis inflorescence stem expressing GUS gene under the control of *NST3* promoter. Bars indicate 100 µm. (d) Quantitative RT-PCR analysis of the gene expression of NST3, SND2, and MYB63 in different cell types of inflorescence stem. The relative expression level of each transcription factor genes was normalized with average expression of *UBQ1*, *ACT2*, and *PP2AA3*. Error bars represent SE of three technical replicates. ND: Not detected.

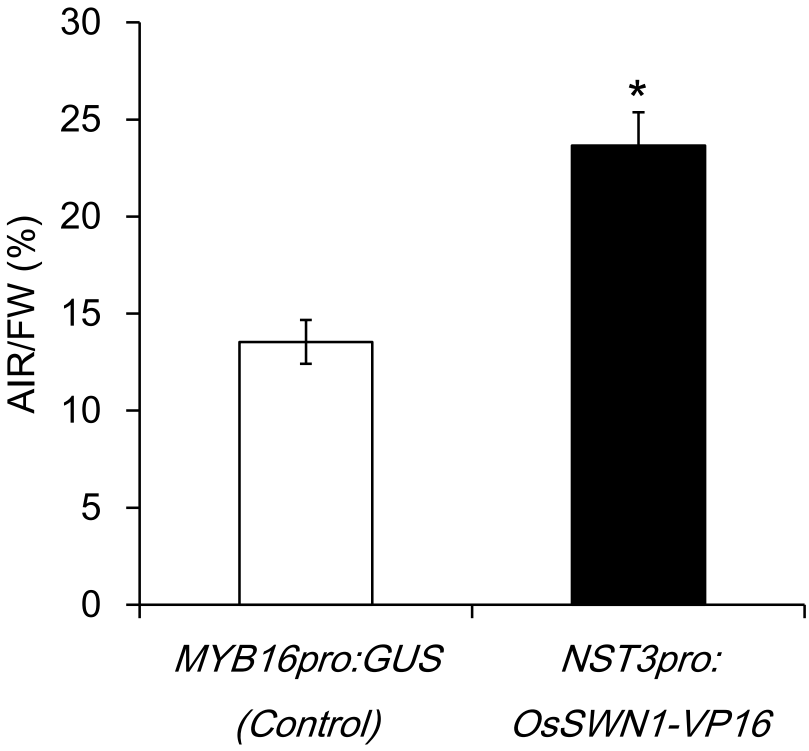

Figure S3. AIR/FW of *NST3pro:OsSWN1-VP16 Arabidopsis* stem.
Error bars represent SD for four biological replicates. Asterisk indicates $P < 0.05$.

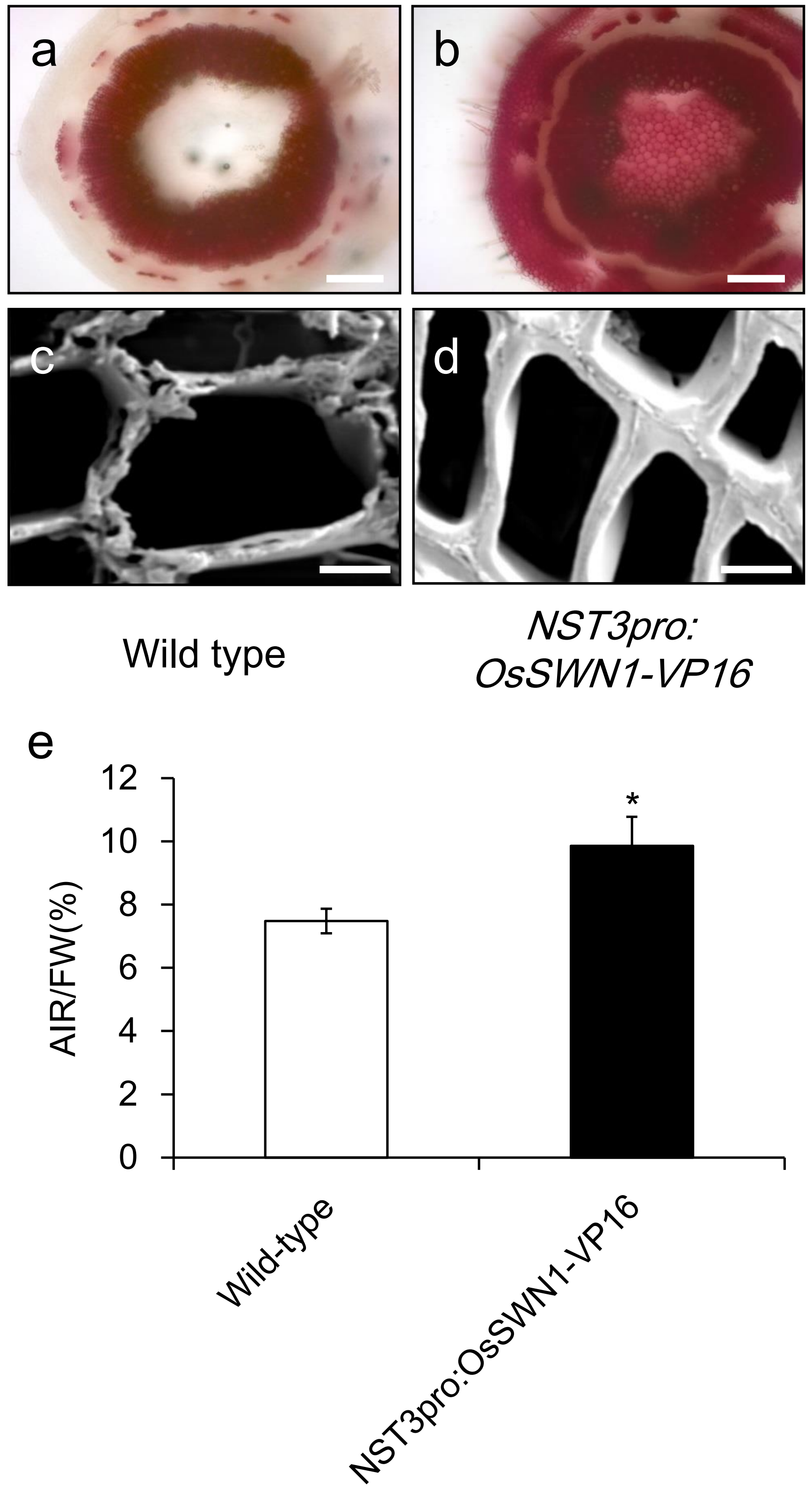

Figure S4. *NST3pro:OsSWN1-VP16* enhanced wood accumulation even in poplar young seedling.
(A, B) Cross section of young stem of wild type (A) and the *NST3pro:OsSWN1-VP16* (B) stained with phloroglucinol. (C, D) SEM image of the cross section of young stem of wild type (C) and the *NST3pro:OsSWN1-VP16* (D). Bars represent 200 µm (A, B) and 4 µm (C, D), respectively. (E) AIR/FW of young stem of wild type (n=3) and the *NST3pro:OsSWN1-VP16* (n = 5). Error bars represent SD of biological replicates of three wild-type and five transgenic poplar. Asterisk indicates $P < 0.05$.

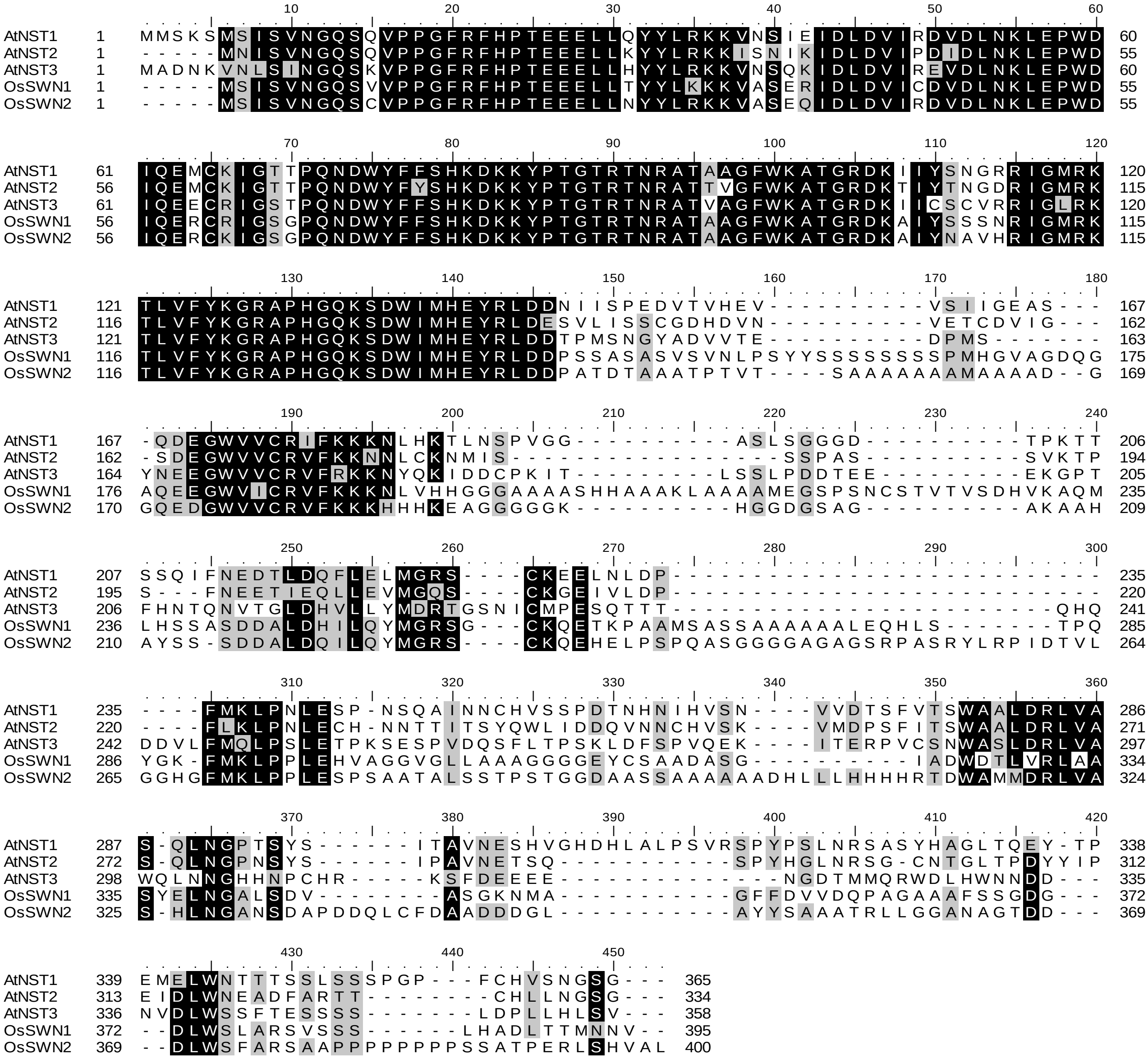

Figure S5. Amino-acid alignment of Arabidopsis NSTs and rice orthologues.
